# Outcomes of catheter intervention for acute pulmonary emboli in a tertiary United Kingdom centre with an established Pulmonary Embolism Response Team (PERT)

**DOI:** 10.1186/s42155-026-00699-3

**Published:** 2026-05-20

**Authors:** Sajal Patel, Narayanan Thulasidasan, Christopher Charles Booth, Amit Gupta, Boris Lams, Karen Breen, Narayan Karunanithy

**Affiliations:** 1 Department of Interventional Radiology, Guy’s & St. Thomas’ NHS Foundation Trust, London, UK; 2 Department of Respiratory Medicine, Guy’s & St. Thomas’ NHS Foundation Trust, London, UK; 3 Thrombosis and Haemophilia Centre, Guy’s & St. Thomas’ NHS Foundation Trust, London, UK; 4 School of Biomedical Engineering and Imaging Sciences, King’s College London, London, UK

**Keywords:** Risk stratification, Thrombolysis, Acute pulmonary embolus, Catheter-directed, PERT, Chronic thromboembolic pulmonary hypertension

## Abstract

**Objective:**

Determine the outcome of patients treated with catheter-directed interventions as per published recommendations for management escalation by the Pulmonary Embolism Response Team (PERT) Consortium.

**Design:**

Retrospective, observational cohort study.

**Materials and methods:**

Retrospective review of patient records managed with catheter-directed intervention between April 2012 and March 2022. Risk stratification was performed as per European Society of Cardiology (ESC) guidelines. Patient demographics, clinical status, and imaging on presentation, procedural details, and outcomes with a minimum follow-up period of 1 year were analysed.

**Results:**

Seventy-nine cases were performed in 76 patients (*n* = 76; mean age 52 years, range 7–86; male:female = 37:39). Fifty-four patients were high-risk, 16 intermediate-high and 7 intermediate-low risk (*n* = 77 cases). 39% of high-risk and 22% of intermediate-risk cases had an absolute or relative contraindication to thrombolytic therapy. Seventeen percent of high-risk and 4% of intermediate-risk cases had a failed trial of systemic thrombolysis. 54% of high-risk and 78% of intermediate-risk cases had a failed trial of anticoagulation. There was a statistically significant reduction in the RV:LV ratio (*p* = 0.05) and clot burden (*p* < 0.0001) following catheter intervention. Available echocardiographic data demonstrated a trend towards improving right heart strain. Bleeding events occurred in 18% of cases, with 79% being high-risk. There was a significant improvement in functional outcomes as per WHO functional status (*p* < 0.001).

**Conclusion:**

Catheter-directed thrombolysis under the guidance of a PERT is a safe and effective therapy and provides a valuable management option for patients who have a contraindication to systemic thrombolysis or have failed a trial of systemic therapy.

**Supplementary Information:**

The online version contains supplementary material available at 10.1186/s42155-026-00699-3.

## Background

Pulmonary embolism (PE) is the third-highest cause of cardiovascular death after myocardial infarction and stroke [1]. Mortality from acute PE can be as high as 50% and it is therefore imperative to identify those at high risk of clinical deterioration and swiftly commence appropriate treatment [2]. Several clinical, biochemical and radiological parameters have been validated to identify acute PE patients at risk of haemodynamic decompensation. The European Society of Cardiology (ESC) is the most widely used risk stratification tool and incorporates many of these parameters [2]. The guideline recommends risk stratification of acute PE into high, intermediate-high, intermediate-low and low-risk categories.

Traditionally, patients with acute PE were anticoagulated, and only given systemic thrombolysis (ST) if in extremis or deemed at high risk of imminent haemodynamic collapse. However, several disease states that may precipitate a PE are also absolute or relative contraindications to ST, including recent major surgery, trauma or intracerebral metastases. The introduction of catheter-directed thrombolysis (CDT) has expanded the group of patients who can be treated with thrombolytics; by using a much smaller volume of thrombolytic agent delivered directly to the clot, patients with a relative contraindication to ST who are deteriorating or failing to improve on anticoagulation have a further treatment escalation option [3–7]. CDT devices currently comprise of either multi-side-hole catheters designed to slowly infuse thrombolytic agent directly into thrombus, or ultrasound-assisted CDT (UACDT) where in addition to a thrombolytic infusion, an ultrasound-emitting core filament within a proprietary infusion catheter delivers ultrasound waves to assist in fibrin matrix unwinding to expose more surface area for the thrombolytic agent to bind [8].

The addition of mechanical thrombectomy (MT) as an adjunctive therapy to CDT allows for on-table thrombus burden reduction and reperfusion of the pulmonary arterial vascular bed which can help immediately alleviate acute right strain and the associated morbidity [9–11]. Used in isolation, MT offers a treatment option for those who have an allergy to thrombolytics or in whom an absolute contraindication prohibits the use of any thrombolytics whatsoever.

Although randomised controlled trial data is emerging [12, 13], at present there is clinical equipoise regarding the efficacy of catheter-directed therapies in PE and therefore considering the elevated mortality risk in the intermediate-high/high-risk groups of patients, specialist input in early decision making is imperative to ensure the best outcome. The concept of a Pulmonary Embolism Response Team (PERT) arose in the USA and aims to provide early multidisciplinary input to high-risk and intermediate-high risk PE patients. The implementation of a PERT has been shown to reduce 30-day mortality rates in both high-risk and intermediate-high risk patients [14].

In this retrospective study, we aim to demonstrate the clinical and functional outcomes of CDT and MT administered under the supervision of a PERT. We also investigate whether any correlation exists between clinical, radiological, and biochemical signs of instability, PE severity, and thrombolytic requirements.

## Methods

### Study design overview

This was a single-centre, retrospective cohort study of patients with PE treated with CDT with or without adjunctive MT between April 2012 and March 2022. After March 2018, all cases were reviewed by a PERT who made a consensus decision on treatment initiation and therapy endpoints determined by clinical response. A record of all cases was obtained by querying the Radiology Information System; clinical, imaging and procedural details were obtained from the Electronic Patient Record and Picture Archiving Software. Patients who underwent catheter-directed interventions more than once for the same hospital episode of PE had the second intervention excluded from outcome analyses.

### Pre-treatment characteristics

Demographic data for all patients treated including relevant comorbidities and history of venous thromboembolic disease was collected. Clinical data including pre-treatment vital signs, from which the National Early Warning Score (NEWS) was calculated, history of collapse or cardiopulmonary arrest, and blood biochemistry and imaging were used to risk stratify as per ESC guidelines [2]. Pulmonary Embolism Severity Index (PESI) and simplified PESI (sPESI) scores were calculated for each patient. Clinical history pertaining to relevant contraindications or recent anticoagulant therapy was recorded. Right ventricular to left ventricular (RV:LV) ratio was measured from corrected multiplanar reformats obtained from pre- and post-procedural computed tomography pulmonary angiography (CTPA), with clot burden calculated as per the 16-point modified Miller Score (MMS) [15].

### Interventions

**Fig. 1:**
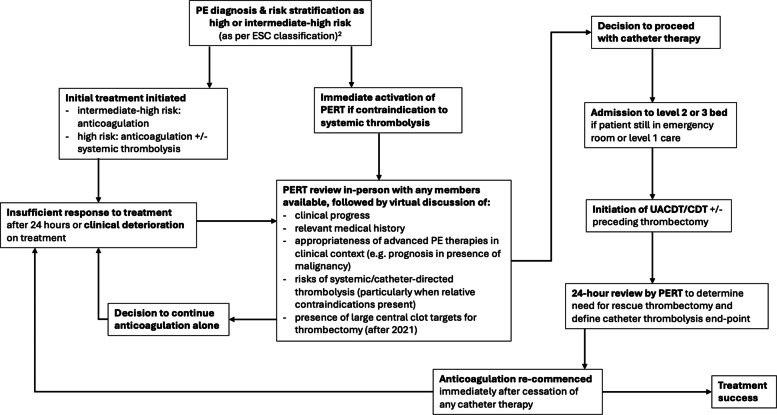
Flowchart demonstrating PERT activation criteria and patient review algorithm

A flowchart demonstrating criteria for PERT activation and decision making is demonstrated in Fig. 1. All procedures were performed by Interventional Radiology, and patients were monitored in a critical care setting. Unless the patient was already intubated, procedures were performed under local anaesthesia. The decision to perform MT was based upon a combination of severity of haemodynamic compromise, presence of central thrombus and availability of a trained operator and equipment, or due to a suboptimal response from local thrombolytic infusion. For CDT/UACDT procedures, unilateral common femoral or internal jugular vein access was obtained and two adjacent 6-French or a single triple-lumen 12-French sheath placed. The right heart was crossed with a pigtail or Cobra catheter and hydrophilic guidewire, followed by catheterisation of a lower lobe pulmonary artery branch and exchange for the thrombolysis delivery catheter. Catheters and the sheath were sutured in place, and thrombolysis commenced. The thrombolytic agent utilised was alteplase, a recombinant tissue plasminogen activator, administered by means of either the EKOS system (Boston Scientific, Marlborough MA, USA) or Cragg-Macnamara infusion catheter (Medtronic, Minneapolis MN, USA). The infusion protocol changed partway through the data collection period, from a dose of 0.5 mg/h per catheter to 1 mg/h per catheter, with exceptions made on a case-by-case basis considering bleeding risk. Fibrinogen levels were measured every 12 h to assess clotting ability, and cryoprecipitate was administered prophylactically when it fell below 0.8 g/L. When performed, adjunctive MT utilised either a 6-French AngioJet catheter (Boston Scientific, Marlborough MA, USA) or the Indigo CAT8 X-TORQ (Penumbra Inc., Alameda, CA, USA). Treatment was terminated based on multidisciplinary assessment of clinical response.

### Outcomes

Clinical and functional outcomes up to at least 1 year were obtained. All available post-treatment CTPA imaging was reviewed to assess clot burden and right heart strain, and post-treatment echocardiogram reports were queried for markers of cardiac function. Performance status data was obtained from EPR documentation including clinic letters and medical notes.

### Statistical analyses

Microsoft Excel and GraphPad Prism were used for data collection and analysis. Descriptive statistics are presented as mean/median/range. Correlation of normally distributed, parametric data was assessed with a Pearson correlation test or a paired *t*-test. Correlation of non-parametric and non-normal data was assessed with Spearman’s correlation test and Wilcoxon signed-rank test; multivariate analyses were performed with a Friedman test. Tests were two-tailed unless stated otherwise, and “*n*” refers to cases unless stated otherwise.

## Results

Seventy-nine cases of catheter-directed treatment of pulmonary embolism (PE) were performed in 76 patients in the study period. Baseline patient characteristics are demonstrated in Table 1. Serum haematological/biochemical markers are demonstrated in Supplemental Table S1. Of the 73 cases for whom data was available regarding initial symptom onset on admission to hospital, 7 were already inpatients having been primarily admitted with non-PE pathology. 70% of cases had experienced haemodynamic instability (defined as any one or more of cardiac arrest, peri-arrest, loss of consciousness and systolic blood pressure of less than 100 mmHg). Of 75 cases with both index CTPA and biochemical markers available, 65 had imaging features and 69 had biochemistry indicating right heart strain (elevated troponin and/or bone natriuretic peptide (BNP)).

**Table 1:** Baseline patient characteristics

Demographics (*n* = 76 patients)		
Age	mean 52 years (range 7–86 years)	
Gender	37 male, 39 female	
Ethnicity	White	43
	Black	14
	Asian	3
	Not recorded	16
Comorbidities	BMI > 30	30
	Hypertension	16
	History of deep vein thrombosis	15
	History of pulmonary embolus	9
	Active cancer	20
	Other prothrombotic state	31
Clinical presentation (*n* = 79 cases)		
	Median time from symptom onset to treatment	3 days (range 0–50)
	Median time from hospital admission to treatment	2 days (range 0–50)
(*n* = 79) (*n* = 73)	PE-related cardiac arrest	12
	PE-related collapse/loss of consciousness	18
	Continuous inotropic requirement	19
(*n* = 77)	ESC high-risk	54
	ESC intermediate-high risk	16
	ESC intermediate-low risk	7
(*n* = 74)	Self-ventilating	51
	Ventilated	11
	Extra-corporeal membrane oxygenation (ECMO)	9
	Prior systemic thrombolysis	10
	Absolute contraindication to systemic thrombolysis	16
	Relative contraindication to systemic thrombolysis	10
	Failed trial of intravenous anticoagulation infusion	33
	Failed trial of oral/subcutaneous anticoagulation	14

The mean PESI score was 112 (range 48–187; *n* = 75) and mean sPESI score was 2 (range 0–4; *n* = 75). The sPESI score was strongly correlated with the NEWS (Spearman *r *= 0.6, *p* < 0.0001, *n* = 70) and moderately correlated with the presence of haemodynamic instability (*r* = 0.4, *p* = 0.0007, *n* = 75). The sPESI score was only weakly correlated with serum troponin (*r* = 0.3, *p* = 0.0086, *n* = 69), and no correlation between the sPESI and the RV:LV ratio (*r* = 0.08, *p* = 0.5), MMS (*r* = 0.12, *p* = 0.3) or BNP (*r* = − 0.01, *p* = 0.9) was seen.

Six cases underwent CDT, 66 underwent UACDT and 3 further cases involved a combination of CDT and UACDT. Large-bore mechanical thrombectomy devices with dedicated PE application were not available for general use in our institution during the study period. One CDT case received adjunctive MT with the Penumbra device, and 4 EKOS cases received adjunctive thrombectomy, 1 with AngioJet and 3 with Penumbra. Sixty-nine cases (87%) had bilateral treatment and 10 underwent unilateral therapy. The median dose of alteplase administered per treatment was 33 mg (*n* = 68; range 4–106 mg) with median infusion duration of 24 h (*n* = 72; range 2–72 h). In 4 cases thrombolytic treatment was stopped early due to complications (3 bleeding events and 1 ischaemic stroke). Adverse events are demonstrated in Table 2; by International Society on Thrombosis and Haemostasis (ISTH) criteria [16], there were 14 cases of major bleeding within 7 days of initiation of thrombolytic treatment, an overall incidence of 18%. Notably, 71% of bleeding events occurred in patients with serum fibrinogen > 1 g/L. Details of other serious adverse events and non-PE related mortalities are demonstrated in Supplemental Table S2.

**Table 2:** Adverse events

Time following initial catheter-based PE treatment	0–7 days	7–30 days	30 days–3 months	3–6 months
International Society on Thrombosis and Haemostasis (ISTH) major bleeding	14	1	0	1
Ischemic stroke	2	3	1	0
Haemorrhagic stroke	1	0	0	0
Other serious adverse events	0	7	0	1

In this predominantly high-risk cohort, all-cause mortalities at 7 and 30 days were 6.6% and 13.2% respectively, incorporating PE-related mortalities of 1.3% and 6.6%. At 30 days, freedom from cardiorespiratory decompensation/collapse was 90.8%, and freedom from requiring extracorporeal membrane oxygenation (ECMO) or institution of mechanical ventilation 96.1%. Pertinent outcome measures out to 3 months, as well as PE recurrence and all-cause mortality to 12 months are detailed in Table 3. Excluding patients initially admitted for clinical reasons other than PE, those who underwent more than one catheter-directed PE treatment during a single admission and those who died within the same admission (*n* = 48 patients fitting these criteria), the median intensive care unit stay was 4 days (mean 7 days; range 1–33 days) and median total hospital stay was 10 days (mean 16 days; range 3–80 days). World Health Organisation (WHO) performance status was recorded at 30 days, 6 and 12 months in *n* = 50 patients (median performance status 1, 1 and 0 respectively), with the improvements significant between 30 days and 6 months and 30 days and 12 months (both *p* < 0.001, Wilcoxon signed-rank test).

**Table 3:** Relevant outcome measures

Time following initial catheter-based PE treatment	0–7 days	7–30 days	30 days–3 months	3–6 months	6–12 months
*n* cases remaining in analysis	79	68	57	54	51
Non-fatal symptomatic confirmed PE recurrence	3	2	0	1	1
Catheter reintervention for PE	2	0	1	0	0
PE-related mortality	1	4	0	0	0
Other mortality	4	1	1	0	1
Cardiorespiratory decompensation/collapse	4	3	0		
Placement on ECMO or mechanical ventilation	1	2	0		

Pre-operative CTPA was available for 75 cases, with a mean RV:LV ratio of 1.6 (range 0.4–3.1) and mean MMS of 10 (range 0–16). By 7 days post-treatment, 20 cases had a repeat CT (at median 2 days, mean 2.8 days, range 1–7 days), with a significant reduction in RV:LV ratio (mean of differences − 0.23, 95% confidence interval − 0.459 to − 0.004; *p* = 0.05) and MMS (median of differences − 5, *p* < 0.0001). Thirty-nine cases had post-procedural imaging at any time-point (median 82 days, mean 230.5 days, range 1–1823 days), demonstrating a statistically significant reduction in RV:LV ratio and MMS (Wilcoxon signed rank test both *p* < 0.0001). Pre- and post-procedural echocardiography data is demonstrated in Table 4, with significant improvements seen in right ventricle basal diameter, left ventricle end-diastolic diameter, right atrium size, tricuspid annular plane systolic excursion and systolic pulmonary arterial pressure.

**Table 4:** Pre- and post-treatment echocardiography

	Right ventricle basal diameter (mm)		Left ventricle end-diastolic diameter (mm)		Right atrium size (cm^2^)		Tricuspid annular plane systolic excursion (cm)		Tricuspid regurgitation maximum velocity (m/s)		Systolic pulmonary arterial pressure (mmHg)		Inferior vena cava diameter (cm)	
	Pre	Post	Pre	Post	Pre	Post	Pre	Post	Pre	Post	Pre	Post	Pre	Post
Median	47	37	38	44	21	16	1.75	1.9	3.7	2.66	43	28	2.1	2.3
(*n*)	(25)	(47)	(25)	(42)	(21)	(30)	(28)	(53)	(9)	(12)	(26)	(36)	(13)	(11)
*p* value	< 0.0001		0.01		0.0058		0.0148		0.1858		0.0029		0.988	

## Discussion

Under the supervision of a PERT, we treated a predominantly high-risk cohort mostly with UACDT and found an encouraging 30-day PE-related mortality of 6.6% with an ISTH-criteria major bleeding rate of 18% within 7 days of treatment. Direct comparison of our cohort with previously published data is difficult due to the heterogenous risk profile and variable reporting of outcomes. A contemporary single-centre German series [17] of 51 intermediate-high and high-risk PE cases treated with UACDT reported a 3.9% in-hospital mortality, with the deaths attributed to pneumonia-associated sepsis rather than directly to the incident PE. A breakdown of risk stratifications was not provided, but it may be assumed that this cohort had less severe disease as the mean sPESI score was 1.3 compared to 2 in our series. At the other end of the risk scale, Dumantepe et al. reported a 90-day mortality of 24.1% in 29 high-risk PE patients who had suffered cardiac arrest and were treated with UACDT as an adjunct to ECMO [18]. The KNOCOUT PE registry [19] included 489 intermediate-high and high-risk patients from 64 international sites treated with UACDT and reported best-in-class results: 30-day all-cause mortality was 1% (despite 5.3% of patients being classified as high-risk, none of the mortalities were from this subgroup) and major bleeding rate in the first 72 h post-treatment was 1.6%. An analysis from the Truveta data platform (which analyses electronic health record data from 16 health systems serving almost a quarter of the population of the USA) focused on bleeding rates from UACDT compared to MT, reporting ISTH major bleeds in 12.4% of UACDT patients and 17.3% in MT [20]. Although risk stratification data and 30-day PE-related or all-cause mortalities could not be obtained from this population-level analysis, the in-hospital mortality was 2.6% in the UACDT cohort.

Regarding technical outcomes, the degree of improvement in right heart strain on imaging from our cohort (a reduction of 0.23 in RV:LV ratio within 7 days of the procedure) was less pronounced than in several of the landmark PE UACDT trials; 0.3 in ULTIMA [21], 0.42 in SEATTLE II [22], 0.35–0.48 between the four arms of OPTALYSE PE [23] and 0.37 in SUNSET sPE [24]. This likely reflects the increased proportion of high-risk patients within our series, and thus the greater severity of right heart dysfunction which may require longer to normalise post-treatment. As we did not routinely perform a 48-h post-treatment CTPA, thrombus burden reduction cannot be accurately compared between our series (which essentially demonstrated a 50% reduction in median MMS within the first 7 days post-treatment) and these earlier EKOS studies. OPTALYSE PE reported a 6–26% reduction in thrombus burden (confounded by some patients being administered rescue systemic thrombolysis) [23] and SUNSET sPE reported a 21% reduction in MMS in the UACDT group at 48 h [24]. Encouragingly, we were able to demonstrate a persistent significant reduction in both right heart strain and clot burden into the medium term, proving treatment efficacy beyond the acute period which is critical in the prevention of chronic thromboembolic pulmonary hypertension [25].

A key area of applicability of our findings is in the treatment of high-risk and intermediate-high-risk patients with absolute or relative contraindications to ST. Conflictingly, risk factors for PE such as active malignancy or recent major surgery (often with consequent immobilisation and/or rapid fluid shifts) often appear as exclusion criteria from major clinical trials for PE therapy. Therefore, the management of these patients in everyday practice cannot be guided from the available evidence base. For example, the SEATTLE II study excluded patients who had suffered a cerebrovascular accident, head trauma, or any other active intracranial or intraspinal disease within the prior 12 months, major surgery within a week or any recent active bleeding from a major organ [22] whereas several patients with one of these characteristics appeared in our cohort. A more “real-world” representation of bleeding risk could be the investigator-initiated and university-sponsored PERFECT trial, which included 9 (out of a total of 101) patients with absolute contraindications to systemic tissue plasminogen activator (of whom eight were treated with CDT and one with MT alone) [26]. PERFECT reported a major bleeding rate of zero, although it should be noted that the in-hospital mortality over a mean hospital stay of 8.23 days was 5.9% (compared to a 6.3% 7-day mortality in our series) despite high-risk PE comprising only 28% of patients compared to 70% in our series. Interestingly, the relationship of PE severity to bleeding risk is further supported by the fact that SEATTLE II patients with massive PE were over three times more likely (23% vs. 7%) to experience major bleeding than those with submassive PE [22], mirroring the findings from our cohort where 79% of the bleeding events occurred in the high-risk group. The paradox of a thrombotic disease process coupled with elevated bleeding risk further underlines the complexity of treating high-risk PE and signposts the need for multidisciplinary input to balance these that a PERT (such as the one at our institution—the first of its kind in the United Kingdom) provides. Our PERT consists of clinicians from haematology, intensive care, respiratory, cardiac surgery and interventional radiology. The PERT opinion was available to any clinician treating intermediate-high-risk or high-risk PE, cases of non-response to therapy, deterioration while on therapy or in cases of contraindications to anticoagulation. PERT input may allow more patients to receive a potentially lifesaving, minimally invasive therapy by facilitating discussions regarding the risks and benefits of anticoagulation, ST and other realistic treatment options in the acute setting. Rosovsky et al. reviewed their patient outcomes before and after the initiation of a PERT, finding that PERT-directed treatment resulted in more high-risk patients being treated with advanced therapies, in particular CDT, with no increase in bleeding events [27]. Continued PERT input also allows for swift changes in treatment strategy as the clinical picture develops which helps prevent prolonged treatment and its inherent risks.

ST remains an important treatment option in hospitals where access to interventional treatments may not be immediately available. However, the bleeding rate is non-negligible and efficacy may vary; one randomised controlled trial comparing ST with heparinisation included only intermediate-risk patients and reported a bleeding rate of 1%, however, 10% of patients required further escalation of treatment [4]. The subsequent PEITHO trial traded a reduction in death or haemodynamic decompensation between the anticoagulation-alone and ST groups (5.6% to 2.6%) with an increase in extracranial bleeding and haemorrhagic stroke—this resulted in there being no statistically significant difference in 30-day mortality between the two treatment strategies [7]. A meta-analysis studying predominantly intermediate-risk patients reported a 9% bleeding rate with ST [6]. These risks can be considered representative of ST outcomes within our healthcare system, as a recent UK study looking at outcomes of ST reported a 9.9% moderate-severe bleeding rate and a 42.2% death rate in high-risk patients along with a 7.5% bleeding rate and 6% death rate in intermediate-risk PE [28]; the outcomes of our cohort compare favourably with these, suggesting that a roll-out of access to interventional therapies across the UK may improve outcomes in this patient group.

The bleeding risk associated with thrombolytics has led to an evolution in thrombectomy devices which are now large enough to effectively remove clot from the pulmonary circulation, possibly resulting in quicker normalisation of right heart function. Occasional usage of MT in our cohort was in an adjunctive capacity alongside CDT. Newer studies are exploring the role of MT to replace thrombolysis. PEERLESS randomised 550 intermediate-risk patients to MT using the FlowTriever™ (Inari Medical, Irvine, CA, USA) or CDT and found a lower risk of clinical deterioration and bail-out therapy requirement in the MT group, along with shorter overall hospital and intensive care unit stays [29]. The STORM-PE trial randomised 100 intermediate-high risk patients to receive anticoagulation plus computer-assisted vacuum thrombectomy with the Lightning Flash Aspiration System (Penumbra Inc., Alameda, CA, USA) or anticoagulation alone and reported that MT plus anticoagulation was superior to anticoagulation only in reducing right heart strain and thrombus burden, and improving speed of physiological recovery, with no device-related complications [13]. Data on 90-day functional outcome and long-term clinical follow-up is awaited. The FLAME study recruited 53 high-risk PE patients to FlowTriever™ treatment, reporting an in-hospital all-cause mortality of 1.9% with an 11.3% major bleeding rate [30], which compares favorably to our high-risk cohort (15% and 22% respectively). However, they also reported a 23% device-related complication rate, which was not seen in our predominantly-CDT cohort. Further randomised trial data is forthcoming, comparing MT to anticoagulation (PEERLESS II), UACDT to anticoagulation (HI-PEITHO), and any catheter-based therapy to anticoagulation alone (PE-TRACT). The high-risk patient cohort is however lacking from all of these trials and as we collect more data on the potential superiority of specific devices against anticoagulation, a meta-analysis of these studies enabling cross-device comparison is needed. Whilst awaiting these data, we might conclude that MT is preferable to CDT (especially in high-risk patients) but in view of the possible increased risk of device-related complications, its use might be best limited to high-volume centres, where escalation to bail-out or bridging therapy such as ECMO may also be available. However, with PE treatment potentially undergoing a similar evolution as that seen in myocardial infarction and cerebrovascular accident over the last four decades (i.e. from ST to catheter-based treatments), CDT might offer a technically less demanding interventional treatment option that could be easier to roll out nationwide. The supervision of a PERT might allow leveraging of the endovascular experience from multiple specialties (interventional radiology, cardiology and vascular surgery) to provide emergent treatment at a larger range of facilities.

### Study limitations

As a retrospective, observational study, there is a reliance on completeness of clinical records. Due to the introduction of a new electronic health record during the timeframe of our study, some historical data was not accessible. Secondly, clinical information regarding the presentation, initial imaging, and biochemical results for some patients who were transferred from external hospitals was not fully transcribed into our electronic record. Correspondingly, some of the patients we treated were repatriated to their local institutions for ongoing care, limiting the follow-up data available. Finally, post-treatment imaging was not mandated and therefore only performed as directed by clinical progress, thus limiting the number of direct pre and post-treatment comparisons that could be performed.

## Conclusion

Our retrospective cohort study demonstrates that catheter-directed interventions in acute high-risk and intermediate-risk PE under the supervision of a PERT are relatively safe and effective and may provide efficacious alternative options in patients with contraindications to ST or who have failed other therapies.

## Supplementary Information

Supplementary Material 1: Supplemental Table S1: Pre-treatment serum haematological/biochemical markers. Supplemental Table S2: Details of serious adverse events and non-PE-related mortalities.

## Data Availability

The datasets used and/or analysed during the current study are available from the corresponding author on reasonable request.

## Abbreviations

PE: Pulmonary embolus
ESC: European Society of Cardiology
ST: Systemic thrombolysis
CDT: Catheter-directed thrombolysis
UACDT: Ultrasound-assisted catheter-directed thrombolysis
MT: Mechanical thrombectomy
PERT: Pulmonary Embolus Response Team
NEWS: National Early Warning Score
PESI: Pulmonary Embolism Severity Index
sPESI: Simplified PESI
RV:LV: Right ventricular to left ventricular
CTPA: Computed tomography pulmonary angiography
MMS: Modified Miller Score
BNP: Bone natriuretic peptide
ISTH: International Society on Thrombosis and Haemostasis
ECMO: Extra-corporeal membrane oxygenation
WHO: World Health Organisation
