# Supplementary material for: Outcomes of catheter intervention for acute pulmonary emboli in a tertiary United Kingdom centre with an established Pulmonary Embolism Response Team (PERT)
Source: CVIR Endovasc. 2026 May 20;9:58. doi: 10.1186/s42155-026-00699-3 (PMC13190918; doi:10.1186/s42155-026-00699-3)
Supplement: Supplementary file 1 — Supplementary Material 1: Supplemental Table S1: Pre-treatment serum haematological/biochemical markers. Supplemental Table S2: Details of serious adverse events and non-PE-related mortalities. [file 42155_2026_699_MOESM1_ESM.docx]

Supplemental Table 1: Pre-treatment serum haematological/biochemical markers

|  | **Haemoglobin**  (g/L) | **Platelets**  (x 10⁹/L) | **eGFR** (mL/min/1.73 m²) | **BNP**  (pg/mL) | **Troponin**  (ng/L) | **Lactate**  (mmol/L) |
| --- | --- | --- | --- | --- | --- | --- |
| n patients | 79 | 79 | 76 | 70 | 73 | 66 |
| Mean | 111 |  | 78 |  |  |  |
| Median |  | 200 |  | 2497 | 57 | 1.6 |
| Range | 58-161 | 53-1384 | 17-152 | 22-27265 | 3-6385 | 0.6-14.1 |

Supplemental Table 2: Details of serious adverse events and non-PE-related mortalities

| **Time following initial catheter-based PE treatment** | **0 - 7 days** | **7 - 30 days** | **30 days - 3 months** | **3 - 6 months** |
| --- | --- | --- | --- | --- |
| Serious adverse events |  | - multiorgan failure - bowel ischaemia, lung abscess - progression of malignancy due to delay in treatment - persistent double vision (peri-ocular bruising) - haemothorax and bronchopleural fistula - acute kidney injury - ventilator associated pneumonia |  | - fasciotomy lower limb |
| Non-PE-related mortality | - intraventricular bleed (recent neurosurgery) - bowel ischaemia - malignant middle cerebral artery territory infarct - middle cerebral artery territory infarct with haemorrhagic transformation | - endocarditis and lung abscess | - metastatic colorectal cancer |  |
